# Impact of Adjuvant Chemotherapy on Breast Cancer Survival: A Real-World Population

**DOI:** 10.1371/journal.pone.0132853

**Published:** 2015-07-27

**Authors:** Lea Rossi, Denise Stevens, Jean-Yves Pierga, Florence Lerebours, Fabien Reyal, Mathieu Robain, Bernard Asselain, Roman Rouzier

**Affiliations:** 1 Institut Curie, Paris, France; 2 Equipe d’Accueil 7285, Risk and safety in clinical medicine for women and perinatal health, University Versailles-Saint-Quentin, Montigny-le-Bretonneux, France; The Norwegian University of Science and Technology (NTNU), NORWAY

## Abstract

**Background:**

The impact of adjuvant chemotherapy on breast cancer prognosis has been demonstrated in randomized trials, but its impact is unknown in real-world populations. The aim of this study was to evaluate the effect of adjuvant chemotherapy on the survival of breast cancer patients in an unselected population.

**Methods:**

This prospective cohort study included 32,502 women treated at the Institut Curie between 1981 and 2008 for a first invasive breast cancer without metastasis. The patients were matched based on their propensity score to receive adjuvant chemotherapy.

**Results:**

The matching generated a subsample of 9,180 patients with an overlapping propensity score. In the group without chemotherapy, the overall survival (OS) rates at 5 and 10 years of follow-up were 87.6% (95% CI [86.7–88.6]) and 75.0% (95% CI [73.6–76.5]), respectively, versus 92.1% (95% CI [91.3–92.9]) and 81.9% (95% CI [80.6–83.2]), respectively, in the chemotherapy group. Distant disease-free survival (DDFS) was significantly improved in the five first years (absolute benefit of 3.5%). In a multivariate analysis, adjuvant chemotherapy was associated with better OS (HR = 0.75, 95% CI [0.69–0.83], p<0.0001) and DDFS (HR = 0.82, 95% CI [0.75–0.90], p<0.0001).

**Conclusion:**

Adjuvant chemotherapy significantly improves OS and DDFS rates in an unselected population, in accordance with previous results reported by randomized trials.

## Introduction

Over the past thirty years, the incidence of breast cancer has greatly increased by 2–3% each year in numerous countries until the mid-2000’s, including several in Europe and North America [1–3]. However, breast cancer mortality has not similarly increased; in fact, it has slightly decreased by approximately 2% each year in most of these countries. This stability and the more recent decline in mortality (despite the increasing incidence rate) reflect an improvement in breast cancer patient survival. This improvement could be explained by treatment advancements in the late 1990s and the increasing proportion of cancers detected at an early stage due to the development of mammographic screening practices.

The advent of chemotherapy most likely contributed significantly to the therapeutic progress observed in the treatment of breast cancer. Indications and prescriptions increased during the 1980s concurrent with the use of cyclophosphamide, methotrexate, and 5-fluorouracil (CMF)-like and anthracycline regimens for patients with metastases. Anthracyclines were later employed in the initial treatment of high-risk breast cancers in the mid-1990s, as were taxane regimens for metastatic patients. Taxanes were used in adjuvant primary treatment in the mid-2000s [4]. Chemotherapy reduced the risk of death due to invasive breast cancers by between 7% and 33% in randomized trials and large meta-analyses; this varied according to tumor characteristics, patient age, and the type and duration of treatment[5]. However, there are very few data on the effects of chemotherapy in real-world populations; additionally, the sparse data for these patients were acquired using limited sub-populations, such as those composed of metastatic patients.

Importantly, an apparent overall effect should not be confused with the actual treatment effect. In experimental conditions, investigators typically achieve this result through the randomization of groups that differ only in treatment allocation [6]. Several methods are feasible for determining the therapeutic effect of chemotherapy, particularly multivariate models and propensity scores [7–8]. These methods are intended to reduce confounding bias and thus clarify the influence attributed to treatment. The propensity score is the probability of being exposed to treatment. This probability is calculated by introducing the main factors that are significantly related to the allocation of treatment into a multivariate regression model. The coefficients for each factor in the regression equation enable score calculation. A propensity score has never been used to determine the impact of breast chemotherapy in real-world populations, with the exception of very specific patient subgroups [9–10].

The aim of this study was to assess the specific impact of chemotherapy on the survival of breast cancer patients in an unselected population.

## Materials and Methods

### Population and data source

Eligible patients included women treated for a primary invasive breast cancer without distant metastasis in the Institut Curie between 1981 and 2008. Patients with a history of previous cancer, with primary chemotherapy, or whose follow-up was less than 3 months were not included.

Data were collected prospectively in this cohort study and were analyzed anonymously. The hospital database was created by knowledgeable workers and validated by a physician. This database was also regularly updated to collect the health status or recent vital status of the patients via phone calls to their doctors and via contacting the governmental records. Data including clinical patient data (age, height, weight, medical history, menopausal status, date of diagnosis, etc.) and tumor characteristics (size, nodal status, Scarff Boom and Richardson (SBR) grade, hormone receptor overexpression (the hormone receptor status was considered positive if the proportion of stained tumor nuclei was ≥10%), human epidermal growth factor receptor-2 (HER2) status, etc.) were gathered from medical records and pathology reports. The treatment sequence was also recorded: dates of surgery, chemotherapy, radiotherapy, hormone therapy or the administration of targeted therapies such as trastuzumab, as well as adjuvant or neoadjuvant administration. Information regarding the recurrence and vital status of the patients was collected and updated every 12 months.

### Study endpoints

Overall survival (OS) was the primary endpoint. We also evaluated the distant disease-free survival (DDFS) and the interval without distant metastasis.

### Statistical analysis

#### Evolution of patients’ characteristics and treatments

To analyze the data by time period, we grouped the dates of diagnosis into 6 periods: a period of 3 years from 1981 to 1983 and 5 periods of 5 years from 1984 to 2008. The analyses of changes in clinical and histological data were performed using the Chi-square test or Fisher’s exact test for qualitative variables and Student's t-test or analysis of variance (ANOVA) for the comparison of means.

#### Survival analysis

The date of diagnosis of breast cancer was considered the start of follow-up. The study cut-off point was November 13^th^ 2013. The OS was measured from the date of diagnosis to the date of death (any cause) or to the date of last contact. DDFS was measured from the date of diagnosis until the date of the distant metastasis of breast cancer or last patient contact. In a univariate analysis, OS and DDFS were assessed using the Kaplan-Meier method, and log-rank tests were used to compare the differences between the resulting curves. Hazard ratios, 95% confidence intervals (CI) and multivariate survival analyses were performed using the Cox proportional-hazards model (adjusted for time period, age, menopausal status, body mass index (BMI), histological type, histological tumor size, histological nodal status, SBR grade and hormone receptor status). A stepwise backward approach was used to select the covariates that were significantly associated with survival.

Primary survival analyses were performed on the population with untreated invasive breast cancer. However, a treatment selection bias was evident in this cohort; this bias was related to multiple factors influencing the decision to administer adjuvant chemotherapy. As a second step, we did a case-control analysis, nested in the cohort. We used a propensity score analysis to minimize the effect of this confounding factor [11]. We derived the propensity score from a logistic regression model using variables associated with the indication of chemotherapy (age, histological type, histological tumor size, histological nodal status, SBR grade, hormone receptor status and time period of treatment) to achieve maximal group similarity for these parameters rather than on the basis of statistical significance. Thus, each patient was assigned a propensity score corresponding to the likelihood of receiving chemotherapy. We matched patients (1 to 1) on their propensity score +/- 0.05 using the ‘‘nearest-neighbor” matching method. We compared the survival of the group treated with chemotherapy with that of the non-treated group (matched using propensity scores).

All statistical tests are reported with two-tailed p-values and 95% CIs at an alpha level of 0.05 or lower. All statistical analyses were performed using R software (http://cran.r-project.org, The R Foundation for Statistical Computing, Vienna, Austria, version 0.98.978, 2009–2013 RStudio, Inc.) with the *rms*, *survival*, and *MatchIt* packages.

#### Ethics Statement

The registration of patients of the Institut Curie in this cohort received a favorable agreement of the french National Committee on Computers and Liberties (CNIL, Commission nationale de l’informatique et des libertes). Patients gave informed written consent prior to be registered in the cohort. The study was approved by the breast cancer study group and the comity of clinical research study of the Institut Curie.

## Results

### Patients characteristics over time

Between January 1981 and December 2008, 48,469 patients were identified in the Institut Curie database. Among these patients, 32,502 women had a previously untreated invasive cancer and met the inclusion criteria. The number of patients increased with time, and their characteristics are detailed in Table 1. The proportion of patients between 50 and 65 years of age increased over time. Grade 1 lesions, hormone receptor positive cancers, and non-palpable (T0) tumors or tumors less than 2 cm (T1) without lymph-node involvement were more prevalent in the most recent periods. The rates of adjuvant chemotherapy, radiotherapy and hormonal therapy increased over time and were applied to a mean of 28.2%, 78.5% and 67.2% of patients, respectively.

**Table 1:** Patient characteristics according to diagnosis periods.

	Years of diagnosis	1981–1983		1984–1988		1989–1993		1994–1998		1999–2003		2004–2008		Total		
		*N*	(%)	*N*	(%)	*N*	(%)	*N*	(%)	*N*	(%)	*N*	(%)	*N*	(%)	*p-value*
	**All**	2331	(7.2)	4401	(13.6)	5886	(18.1)	6407	(19.7)	4912	(15.1)	8565	(26.3)	32502	(100)	.
**Age (years)**	**< 35**	64	(2.7)	102	(2.3)	134	(2.3)	110	(1.7)	69	(1.4)	136	(1.6)	615	(1.9)	<0.0001
	**35 to 49**	685	(29.4)	1229	(27.9)	1604	(27.2)	1666	(26.0)	1058	(21.5)	1919	(22.4)	8161	(25.1)	
	**50 to 65**	883	(37.9)	1896	(43.1)	2590	(44.0)	2930	(45.7)	2342	(47.7)	3901	(45.5)	14542	(44.7)	
	**> 65**	647	(27.8)	1149	(26.1)	1558	(26.5)	1701	(26.5)	1443	(29.4)	2609	(30.5)	9107	(28.0)	
	**NA**	52	(2.2)	25	(0.6)	0	(0)	0	(0)	0	(0)	0	(0)	77	(0.2)	
**Menopausal status**	**post-menopausal**	1287	(55.2)	2523	(57.3)	3323	(56.5)	3331	(52.0)	3198	(65.1)	5577	(65.1)	19239	(59.2)	<0.0001
	**pre-menopausal**	799	(34.3)	1443	(32.8)	1683	(28.6)	1699	(26.5)	1163	(23.7)	1946	(22.7)	8733	(26.9)	
	**NA**	245	(10.5)	435	(9.9)	880	(14.9)	1377	(21.5)	551	(11.2)	1042	(12.2)	4530	(13.9)	
**Clinical tumor size**	**T0**	62	(2.7)	175	(4.0)	564	(9.6)	875	(13.7)	770	(15.7)	1222	(14.3)	3668	(11.3)	<0.0001
	**T1**	636	(27.3)	1505	(34.2)	2097	(35.6)	2590	(40.4)	2446	(49.8)	4980	(58.1)	14254	(43.9)	
	**T2**	1287	(55.2)	2272	(51.6)	2548	(43.3)	2357	(36.8)	1377	(28.0)	1808	(21.1)	11649	(35.8)	
	**T3**	253	(10.8)	269	(6.1)	408	(6.9)	352	(5.5)	159	(3.2)	235	(2.7)	1676	(5.2)	
	**T4**	86	(3.7)	147	(3.3)	257	(4.4)	216	(3.4)	108	(2.2)	111	(1.3)	925	(2.8)	
	**NA**	7	(0.3)	33	(0.7)	12	(0.2)	17	(0.3)	52	(1.1)	209	(2.4)	330	(1.0)	
**Involved axillary nodes**	**0**	1100	(47.2)	2339	(53.1)	3309	(56.2)	3600	(56.2)	2981	(60.7)	5493	(64.1)	18822	(57.9)	<0.0001
	**1 to 3**	535	(22.9)	1028	(23.4)	1169	(19.9)	1420	(22.2)	1186	24.1)	2060	(24.0)	7398	(22.8)	
	**4 to 9**	214	(9.2)	335	(7.6)	429	(7.3)	494	(7.7)	323	(6.6)	550	(6.4)	2345	(7.2)	
	**≥ 10**	91	(3.9)	157	(3.6)	180	(3.1)	191	(3.0)	94	(1.9)	184	(2.1)	897	(2.8)	
	**NA**	391	(16.8)	542	(12.3)	799	(13.6)	702	(11.0)	328	(6.7)	278	(3.2)	3040	(9.3)	
**SBR grade**	**1**	352	(15.1)	1048	(23.8)	1450	(24.6)	1775	(27.7)	1664	(33.9)	2543	(29.7)	8832	(27.2)	<0.0001
	**2**	1027	(44.1)	1828	(41.5)	2136	(36.3)	2126	(33.2)	1904	(38.8)	3682	(43.0)	12703	(39.1)	
	**3**	853	(36.6)	1226	(27.9)	1784	(30.3)	2050	(32.0)	1051	(21.4)	1953	(22.8)	8917	(27.4)	
	**NA**	99	(4.2)	299	(6.8)	516	(8.8)	456	(7.1)	293	(6.0)	387	(4.5)	2050	(6.3)	
**Hormone receptor status**	**Hormone receptor +**	1497	(64.2)	3029	(68.8)	4204	(71.4)	4282	(66.8)	3723	(75.8)	7181	(83.8)	23916	(73.6)	<0.0001
	**Hormone receptor -**	394	(16.9)	689	(15.6)	917	(15.6)	834	(13.0)	562	(11.4)	1108	(12.9)	4504	(13.9)	
	**NA**	440	(18.9)	683	(15.5)	765	(13.0)	1291	(20.1)	627	(12.8)	276	(3.2)	4082	(12.6)	
**Adjuvant chemotherapy**	**no**	2080	(89.2)	3630	(82.5)	4452	(75.6)	4616	(72.0)	3164	(64.4)	5395	(63.0)	23337	(71.8)	<0.0001
	**yes**	251	(10.8)	771	(17.5)	1434	(24.4)	1791	(28.0)	1748	(35.6)	3170	(37.0)	9165	(28.2)	
**Hormone therapy**	**no**	1575	(67.6)	2814	(63.9)	3929	(66.7)	3982	(62.1)	1720	(35.0)	2812	(32.8)	16832	(51.8)	<0.0001
	**yes**	756	(32.4)	1587	(36.1)	1957	(33.3)	2425	(37.9)	3192	(65.0)	5753	(67.2)	15670	(48.2)	
**Radiotherapy**	**no**	1156	(49.6)	1533	(34.8)	1495	(25.4)	1060	(16.5)	741	(15.1)	991	(11.6)	6976	(21.5)	<0.0001
	**yes**	1175	(50.4)	2868	(65.2)	4391	(74.6)	5347	(83.5)	4171	(84.9)	7574	(88.4)	25526	(78.5)	

NA: not assessable. T0: no palpable tumor. T1: clinical tumor size ≤ 2 cm. T2: tumor > 2 cm and ≤ 5 cm. T3: >5 cm. T4: extension to skin and/or chest wall. SBR: Scarff Bloom and Richardson.

The median follow-up was 100 months. Eighty-five per cent of women were followed for at least 5 years or until death. Over the 28 years of the study, 8,119 (25%) deaths and 5,946 distant metastasis (18%) were observed among the 32,502 patients with invasive breast cancer.

### Matching on propensity scores

**Fig 1:**
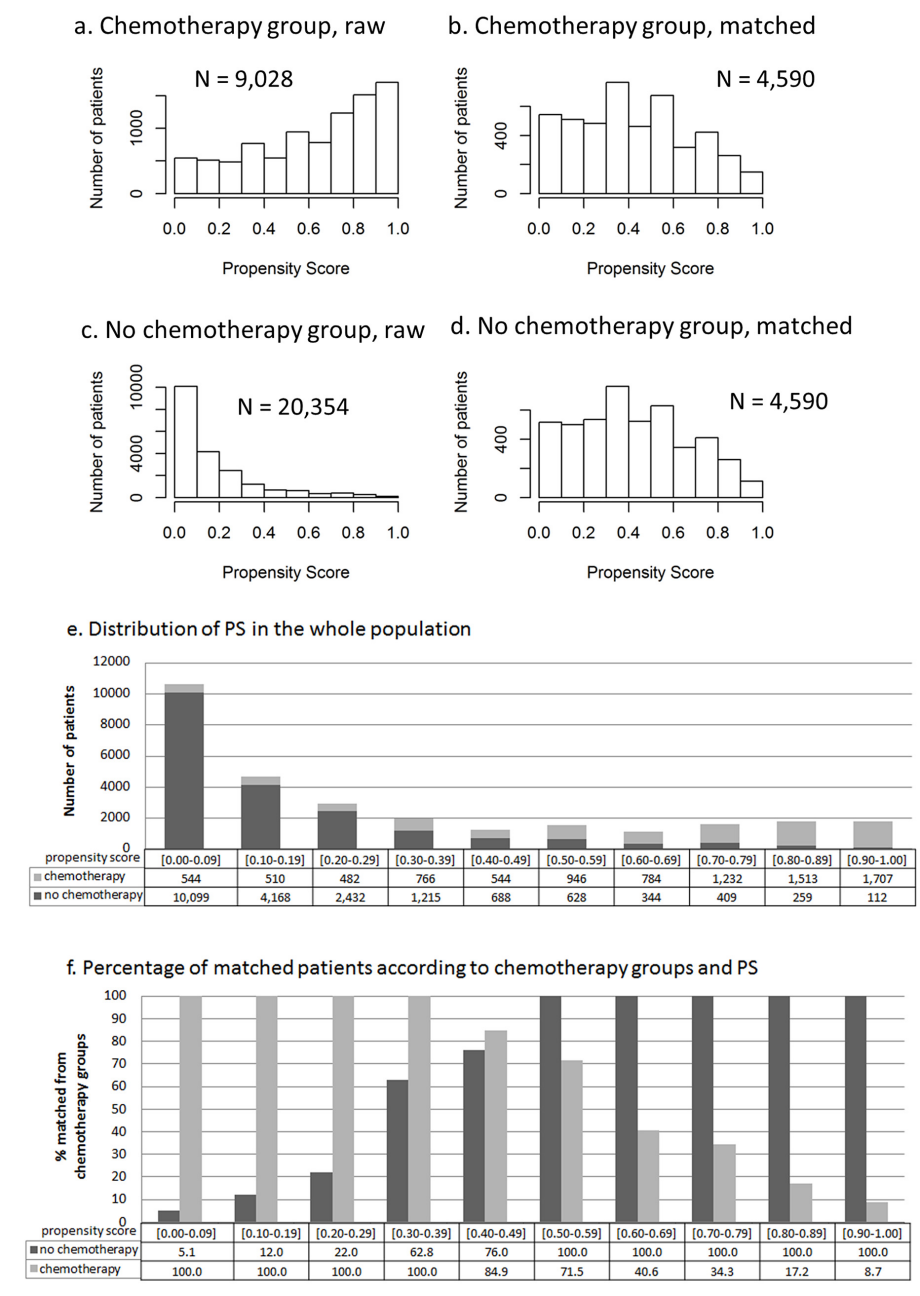
Distribution of propensity scores prior to and after matching. Mean propensity score (PS) before matching was 0.69 (SD = 0.20) in the chemotherapy-treated group and 0.28 (SD = 0.20) in the non-treated group (p<0.0001). After matching, it was 0.43 (SD = 0.25) in the chemotherapy-treated group and 0.42 (SD = 0.25) in the non-treated group (p = 0.22).

From the full sample of 32,502 women with an invasive untreated breast cancer, 29,382 patients without missing data could be included in the model, and a subsample was created composed of 9,180 women with overlapping propensity scores to receive chemotherapy. Propensity score matching improved the similarities in each factor distribution and resulted in overall propensity scores that were not significantly different after matching (Table 2, Fig 1A–1D). This approach resulted in the selection of all patients with low propensity scores who received chemotherapy and all patients with high propensity scores who did not receive chemotherapy (Fig 1E and 1F).

**Table 2:** Characteristics of the chemotherapy and no chemotherapy groups prior to and after matching via propensity scores.

		PRIOR TO MATCHING					AFTER MATCHING				
		no chemotherapy		chemotherapy			no chemotherapy		chemotherapy		
		*N*	(%)	*N*	(%)	*p-value*	*N*	(%)	*N*	(%)	*p-value*
	**All**	23337	(71.8)	9165	(28.2)	.	4590	(50)	4590	(50)	.
**Age (years)**	**< 35**	257	(1.1)	358	(3.9)	<0.0001	102	(2.2)	186	(4.0)	<0.0001
	**35 to 49**	4630	(19.8)	3531	(38.5)		1349	(29.4)	1346	(29.3)	
	**50 to 65**	10172	(43.6)	4370	(47.7)		2274	(49.5)	2281	(49.7)	
	**> 65**	8201	(35.1)	906	(9.9)		865	(18.8)	777	(16.9)	
	**NA**	77	(0.3)	0	(0)		0	(0)	0	(0)	
**Histological type**	**Ductal**	15704	(67.3)	7594	(82.9)	<0.0001	3593	(78.3)	3558	(77.5)	0.68
	**Lobular**	2925	(12.5)	926	(10.1)		549	(12.0)	569	(12.4)	
	**Other**	4708	(20.2)	645	(7.0)		448	(9.8)	463	(10.1)	
**Histological tumor size**	**pT1**	13989	(59.9)	4578	(49.9)	<0.0001	2526	(55.0)	2593	(56.5)	0.01
	**pT2**	4940	(21.2)	3761	(41.0)		1629	(35.5)	1517	(33.0)	
	**pT3**	363	(1.5)	351	(3.8)		133	(2.9)	167	(3.6)	
	**pT4**	45	(0.2)	42	(0.4)		10	(0.2)	22	(0.5)	
	**NA**	4000	(17.1)	433	(4.7)		292	(6.4)	291	(6.4)	
**Involved axillary nodes**	**0**	15885	(68.1)	2937	(32.0)	<0.0001	2218	(48.3)	2207	(48.1)	<0.0001
	**1 to 3**	3420	(14.7)	3978	(43.4)		1706	(37.2)	1526	(33.2)	
	**4 to 9**	826	(3.5)	1519	(16.6)		473	(10.3)	611	(13.3)	
	**≥ 10**	297	(1.3)	600	(6.6)		193	(4.2)	246	(5.3)	
	**NA**	2909	(12.5)	131	(1.4)		0	(0)	0	(0)	
**SBR grade**	**1**	7553	(32.4)	1279	(14.0)	<0.0001	929	(20.2)	1001	(21.8)	0.003
	**2**	9000	(38.6)	3703	(40.4)		1934	(42.1)	2031	(44.2)	
	**3**	4903	(21.0)	4014	(43.8)		1727	(37.7)	1558	(34.0)	
	**NA**	1881	(8.0)	169	(1.8)		0	(0)	0	(0)	
**Hormone receptor status**	**Hormone receptor +**	17424	(74.7)	6492	(70.8)	<0.0001	3349	(75.1)	3446	(75.1)	0.82
	**Hormone receptor -**	2349	(10.0)	2155	(23.5)		763	(16.7)	779	(17.0)	
	**NA**	3564	(15.3)	518	(5.7)		378	(8.2)	365	(7.9)	
**Years of diagnosis**	**1981–1983**	2080	(89.1)	251	(2.7)	<0.0001	160	(3.5)	171	(3.7)	<0.0001
	**1984–1988**	3630	(15.6)	771	(8.4)		516	(11.2)	493	(10.7)	
	**1989–1993**	4452	(19.1)	1434	(15.6)		978	(21.3)	950	(20.7)	
	**1994–1998**	4616	(19.8)	1791	(19.5)		956	(20.8)	1060	(23.1)	
	**1999–2003**	3164	(13.6)	1748	(19.1)		705	(15.3)	818	(17.8)	
	**2003–2008**	5395	(23.1)	3170	(34.6)		1275	(27.8)	1098	(23.9)	
**Propensity score**	**Mean (sd)**	0.28 (0.20)		0.69 (0.20)		<0.0001	0.42 (0.25)		0.43 (0.25)		0.22

NA: not assessable. pT1: pathological tumor size ≤ 2 cm. pT2: tumor > 2 cm and ≤ 5 cm. pT3: > 5 cm. pT4 = extension to skin and/or chest wall. SBR: Scarff Bloom and Richardson. sd: standard deviation.

### OS

**Fig 2:**
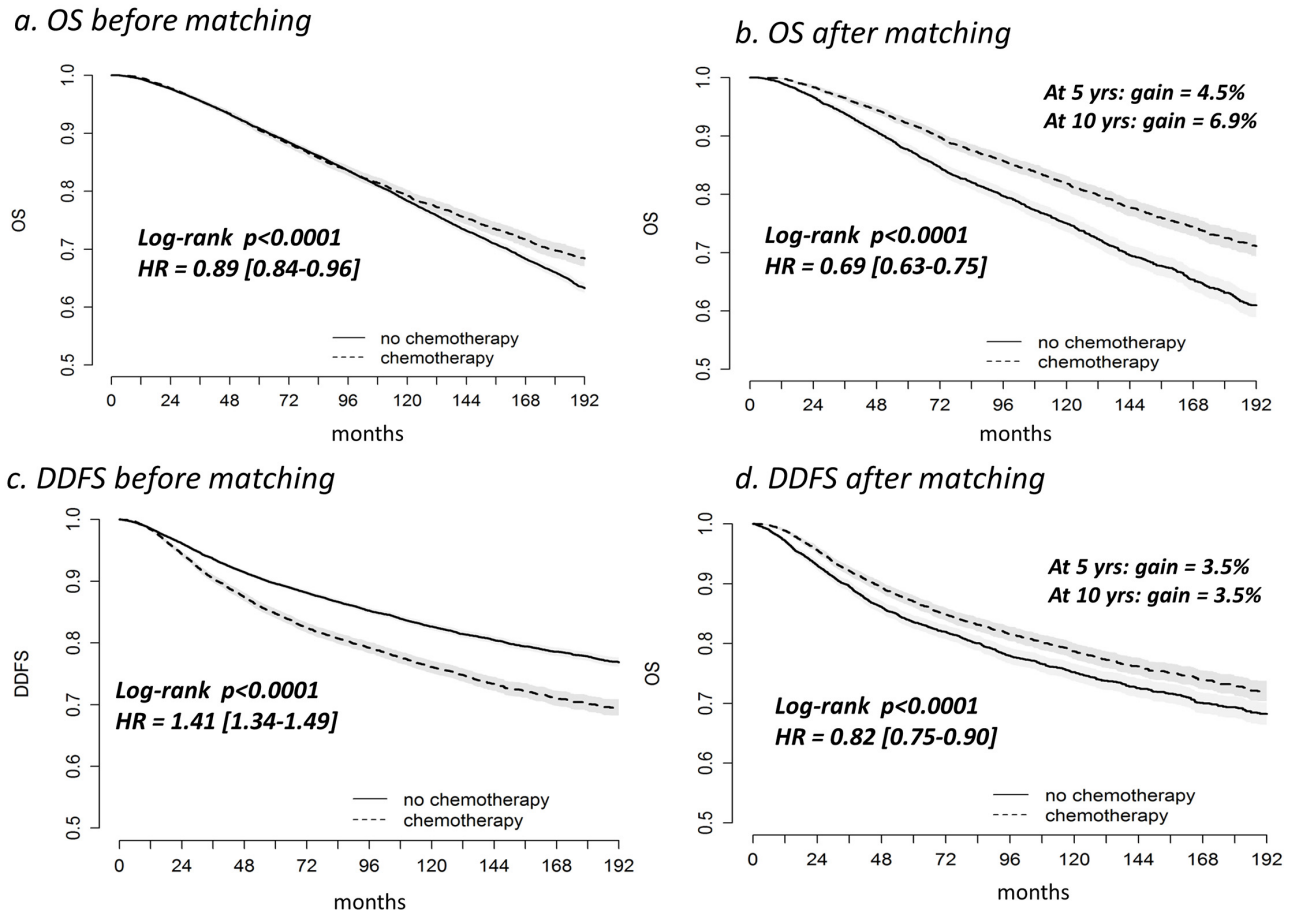
Overall survival (OS) and distant disease-free survival (DDFS) according to adjuvant chemotherapy prior to and after matching via propensity scores. HR: hazard ratio.

In a univariate analysis, the OS of the 9,165 patients who received adjuvant chemotherapy was significantly better than that of the 20,354 patients who did not receive chemotherapy (raw HR = 0.89, 95% CI [0.84–0.96], p<0.0001) (Fig 2, Table 3). However, this significant difference did not reflect the clinical impact of chemotherapy. The patients who received chemotherapy were younger than the patients treated without chemotherapy (Table 2) with better late OS (selection bias). After matching via propensity scores, a univariate analysis showed that the patients who received adjuvant chemotherapy exhibited a better OS than those who did not receive chemotherapy (HR = 0.69, 95% CI [0.63–0.75], p<0.0001). The 5-year survival rate in the patients treated with chemotherapy was 92.1% with a 95% CI [91.3–92.9] versus 87.6% with a 95% CI [86.7–88.6] in the untreated group (absolute benefit: 4.5%). At 10 years, the survival rate in the group treated with chemotherapy was 81.9% with a 95% CI [80.6–83.2] versus 75.0% with a 95% CI [73.6–76.5] in the untreated group (absolute benefit: 6.9%). In multivariate analysis of the matched population, the difference of OS attributable to the adjuvant chemotherapy remained significant (HR = 0.75, 95% CI [0.69–0.83], p<0.0001) (Table 4). The hormone receptor overexpression status, the pathological tumor size, the number of invaded nodes, and the SBR grade were also associated with prognosis.

**Table 3:** Survival prior to matching: prognosis factors in univariate and multivariate analyses.

		OS				DDFS			
		Univariate		Multivariate		Univariate		Multivariate	
		HR [CI 95%]	*p-value*	HR [CI 95%]	*p-value*	HR [CI 95%]	*p-value*	HR [CI 95%]	*p-value*
**Years of diagnosis**	**1981–1983**	1	.	1	.	1	.	1	.
	**1984–1988**	0.85 [0.80–0.91]	<0.0001	0.89 [0.70–1.13]	0.33	0.89 [0.82–0.97]	0.009	1.01 [0.76–1.34]	0.95
	**1989–1993**	0.75 [0.71–0.81]	<0.0001	0.87 [0.69–1.10]	0.25	0.86 [0.79–0.93]	0.0002	0.97 [0.74–1.28]	0.86
	**1994–1998**	0.67 [0.62–0.72]	<0.0001	0.77 [0.61–0.98]	0.03	0.75 [0.69–0.82]	<0.0001	0.82 [0.62–1.08]	0.16
	**1999–2003**	0.51 [0.47–0.56]	<0.0001	0.68 [0.51-.092]	0.01	0.56 [0.51–0.62]	<0.0001	0.61 [0.44–0.84]	0.0002
	**2004–2008**	0.38 [0.35–0.42]	<0.0001	0.42 [0.32–0.55]	<0.0001	0.41 [0.37–0.46]	<0.0001	0.44 [0.32–0.59]	<0.0001
**Age (years)**	**< 35**	1.47 [1.31–1.65]	<0.0001	1.42 [1.00–2.02]	0.05	2.01 [1.88–2.35]	<0.0001	1.64 [1.24–2.16]	0.0005
	**35 to 49**	0.86 [0.82–0.91]	<0.0001	1.15 [0.96–1.37]	0.13	1.21 [1.14–1.27]	<0.0001	1.14 [1.01–1.29]	0.03
	**50 to 65**	1	.	1	.	1	.	1	.
	**> 65**	2.25 [2.15–2.37]	<0.0001	2.06 [1.85–2.30]	<0.0001	1.00 [0.94–1.06]	0.99	1.09 [0.9–1.25]	0.17
**Menopausal status**	**post-menopausal**	1	.	1	.	1	.	1	.
	**pre-menopausal**	0.62 [0.59–0.65]	<0.0001	0.68 [0.57–0.80]	<0.0001	1.23 [1.17–1.29]	<0.0001	.	NS
**BMI (kg/m²)**	**< 18.5**	0.96 [0.83–1.11]	0.57	1.25 [1.02–1.52]	0.03	0.92 [0.79–1.09]	0.38	1.16 [0.93–1.45]	0.20
	**[18.5–25[**	1	.	1	.	1	.	1	.
	**[25–30[**	1.29 [1.20–1.39]	<0.0001	1.13 [1.01–1.25]	0.03	1.18 [1.09–1.27]	<0.0001	1.09 [0.97–1.22]	0.16
	**≥ 30**	1.49 [1.35–1.65]	<0.0001	1.22 [1.05–1.41]	0.008	1.31 [1.18–1.46]	<0.0001	1.22 [1.04–1.43]	0.01
**Histological type**	**Ductal**	1	.	1	.	1	.	1	.
	**Lobular**	1.07 [1.00–1.14]	0.04	.	NS	1.08 [1.00–1.16]	0.04	.	NS
	**Other**	1.05 [0.99–1.10]	0.1	.	NS	0.95 [0.88–1.01]	0.11	.	NS
**Histological tumor size**	**pT1**	1	.	1	.	1	.	1	.
	**pT2**	2.17[2.06–2.28]	<0.0001	1.47 [1.34–1.63]	<0.0001	2.54 [2.39–2.70]	<0.0001	1.66 [1.49–1.84]	<0.0001
	**pT3**	3.18 [2.81–3.59]	<0.0001	2.26 [1.83–2.80]	<0.0001	3.94 [3.44–4.50]	<0.0001	2.20 [1.76–2.75]	<0.0001
	**pT4**	2.53 [1.61–3.97]	<0.0001	1.65 [0.73–3.73]	0.23	2.93 [1.89–4.56]	<0.0001	2.34 [1.27–4.29]	0.006
**Involved axillary nodes**	**0**	1	.	1	.	1	.	1	.
	**1 to 3**	1.54 [1.47–1.63]	<0.0001	1.22 [1.09–1.36]	0.0006	1.96 [1.85–2.08]	<0.0001	1.47 [1.31–1.67]	<0.0001
	**4 to 9**	2.94[2.76–3.13]	<0.0001	2.14 [1.85–2.48]	<0.0001	4.47 [4.18–4.78]	<0.0001	2.93 [2.52–3.42]	<0.0001
	**≥ 10**	4.81 [4.43–5.22]	<0.0001	3.38 [2.75–4.15]	<0.0001	7.75 [7.12–8.44]	<0.0001	5.52 [4.54–6.72]	<0.0001
**SBR grade**	**1**	1	.	1	.	1	.	1	.
	**2**	1.56 [1.47–1.66]	<0.0001	1.09 [0.96–1.24]	0.20	2.16 [2.00–2.33]	<0.0001	1.61 [1.37–1.89]	<0.0001
	**3**	2.41 [2.27–2.56]	<0.0001	1.84 [1.63–2.08]	<0.0001	3.69 [3.43–3.98]	<0.0001	2.68 [2.31–3.12]	<0.0001
**Hormone receptor status**	**Hormone receptor +**	1	.	1	.	1	.	1	.
	**Hormone receptor -**	1.55 [1.47–1.64]	<0.0001	1.34 [1.18–1.52]	<0.0001	1.65 [1.56–1.74]	<0.0001	1.26 [1.10–1.44]	0.0007
**Adjuvant chemotherapy**	**No**	1	.	1	.	1	.	1	.
	**Yes**	0.89 [0.84–0.96]	<0.0001	0.88 [0.78–1.00]	0.05	1.41 [1.34–1.49]	<0.0001	0.85 [0.74–0.97]	0.01

OS: overall survival. DDFS: Distant disease-free survival. BMI: body mass index. pT1: pathological tumor size ≤ 2 cm. pT2: tumor > 2 cm and ≤ 5 cm. pT3: > 5 cm. pT4: extension to skin and/or chest wall. SBR: Scarff Bloom and Richardson. HR: hazard ratio. NS: not significant in multivariate analysis and therefore not included in the final multivariate model.

**Table 4:** OS and DDFS after matching: prognosis factors in multivariate analysis.

		OS		DDFS	
		HR [CI 95%]	*p-value*	HR [CI 95%]	*p-value*
**Years of diagnosis**	**1981–1983**	1	.	1	.
	**1984–1988**	0.92 [0.77–1.09]	0.33	0.98 [0.82–1.18]	0.84
	**1989–1993**	0.81 [0.68–0.97]	0.020	0.93 [0.78–1.11]	0.43
	**1994–1998**	0.75 [0.63–0.90]	0.002	0.79 [0.66–0.95]	0.01
	**1999–2003**	0.56 [0.46–0.72]	<0.0001	0.64 [0.52–0.80]	<0.0001
	**2004–2008**	0.37 [0.29–0.48]	<0.0001	0.46 [0.36–0.58]	<0.0001
**Age (years)**	**< 35**	1.32 [0.99–1.76]	0.05	1.50 [1.20–1.88]	0.0004
	**35 to 49**	0.97 [0.82–1.15]	0.73	1.09 [0.98–1.21]	0.12
	**50 to 65**	1	.	1	.
	**> 65**	1.64 [1.45–1.85]	<0.0001	1.04 [0.92–1.18]	0.52
**Menopausal status**	**post-menopausal**	1	.	1	.
	**pre-menopausal**	0.79 [0.67–0.93]	0.004	.	NS
**BMI (kg/m²)**	**< 18.5**	1	.	1	NS
	**[18.5–25[**	.	NS	.	.
	**[25–30[**	.	NS	.	NS
	**≥ 30**	.	NS	.	NS
**Histological type**	**Ductal**	1	.	1	.
	**Lobular**	.	NS	.	NS
	**Other**	.	NS	.	NS
**Histological tumor size**	**pT1**	1	.	1	.
	**pT2**	1.43 [1.29–1.59]	<0.0001	1.59 [1.44–1.77]	<0.0001
	**pT3**	1.54 [1.23–1.92]	<0.0001	1.79 [1.46–2.21]	<0.0001
	**pT4**	1.18 [0.44–3.19]	0.74	1.54 [0.69–3.47]	0.29
**Involved axillary nodes**	**0**	1	.	1	.
	**1 to 3**	1.40 [1.24–1.58]	<0.0001	1.51 [1,34–1.69]	<0.0001
	**4 to 9**	2.56 [2.22–2.94]	<0.0001	3.02 [2.64–3.46]	<0.0001
	**≥ 10**	3.95 [3.34–4.67]	<0.0001	5.41 [4.61–6.35]	<0.0001
**SBR grade**	**1**	1	.	1	.
	**2**	1.48 [1.27–1.74]	<0.0001	1.73 [1.48–2.03]	<0.0001
	**3**	2.00 [1.71–2.35]	<0.0001	2.34 [1.99–2.74]	<0.0001
**Hormone receptor status**	**Hormone receptor +**	1	.	1	.
	**Hormone receptor -**	1.29 [1.15–1.45]	<0.0001	1.24 [1.10–1.39]	0.0003
**Adjuvant chemotherapy**	**No**	1	.	1	.
	**Yes**	0.75 [0.69–0.83]	<0.0001	0.82 [0.75–0.90]	<0.0001

OS: overall survival. DDFS: Distant disease-free survival. BMI: body mass index. pT1: pathological tumor size ≤ 2 cm. pT2: tumor > 2 cm and ≤ 5 cm. pT3: > 5 cm. pT4 = extension to skin and/or chest wall. SBR: Scarff Bloom and Richardson. HR: hazard ratio. NS: not significant in multivariate analysis and therefore not included in the final multivariate model.

### DDFS

Prior to matching, the patients who received adjuvant chemotherapy had a worse DDFS than those who did not receive chemotherapy, as found in a univariate analysis (raw HR = 1.41, 95% CI [1.34–1.49], p<0.0001) (Fig 2, Table 3); this reflected an indication bias (patients with worse prognosis received chemotherapy more often). After matching for propensity scores, the risk of distant metastasis was lower in the patients who were treated with chemotherapy (HR = 0.82, 95% CI [0.75–0.90], p<0.0001), as found in a univariate analysis. At 5 and 10 years of follow-up, the women who were treated with chemotherapy had an absolute benefit of 3.5% with respect to DDFS. In multivariate analysis of the matched population, the HR was 0.82, 95% CI [0.75–0.90] (p<0.0001) (Table 4).

## Discussion

An improvement in patient prognosis was observed as a result of the administration of adjuvant chemotherapy in a real-world setting (32,502 women). After patients were matched based on their propensity score to receive chemotherapy, the treated group in the period between 1981 and 2008 exhibited a 25% reduction in the relative risk of death compared with the untreated group (HR = 0.75 [0.69–0.83], p<0.0001). This reduction resulted in an absolute survival benefit of 4.5% at 5 years and 6.9% at 10 years. Chemotherapy was also associated with an 18% relative reduction in the risk of distant metastasis (HR = 0.82 [0.75–0.90], p<0.0001). The absolute benefit in terms of DDFS was 3.5% at both 5 and 10 years.

Prior to matching, OS was significantly better in the treatment group; this difference appeared on the survival curve at 10 years of follow-up (Fig 2). This result can be explained by the fact that the group that did not receive chemotherapy included many more patients over 65 years of age as well as more patients from earlier periods. Using death from any cause results in the accumulation of events in older patients. This factor explains the better absolute benefit in terms of OS compared with DDFS in favor of chemotherapy after matching. This absolute benefit could also reflect more co-morbidities in patients who did not receive chemotherapy. Matching via propensity score created more comparable groups; however, co-morbidities were not included in the model and were therefore not taken into account when matching. This propensity score approach reduced indication bias [7–8], enabling a more accurate estimate of the effect of chemotherapy. We could not take into account the effects of HER2 status, the type of chemotherapy regimen, the type of hormonal therapy, comorbidities or screening programs because we lacked prospectively collected data for these parameters. Only 1.6% of our entire population received trastuzumab (data not shown).

In 1998, a meta-analysis by the Early Breast Cancer Trialists' Collaborative Group [12] showed that chemotherapy was associated with a relative risk reduction of death that was estimated to be between 8% and 27% depending on the age of the patients and the type of breast cancer (RR = 0.85 in a population of 17,723 patients). The benefit of chemotherapy was independent from menopausal status, lymph node involvement and the use of hormone therapy. This study included trials primarily testing the CMF (cyclophosphamide, methotrexate, 5-fluorouracil) combination. Thus, adjuvant chemotherapy was more widely administered. Later, anthracyclines [13] and taxanes [14] demonstrated superior efficacy [5] and were integrated into chemotherapy regimens. Substantial differences in age and comorbidities have been reported between patients in randomized clinical trials and real-world registries because of explicit exclusion criteria and subtle recruitment biases [15]. However, there are no data on the impact of chemotherapy on breast cancer survival in the real world, with the exception of a few population-based studies reviewing specific sub-groups such as metastatic cancer, HER2+ tumors, triple negative breast cancer or elderly patients [9] [10] [16]. Our results, which were obtained from an unselected population, are consistent with those from randomized controlled trials. In a real clinical setting, older patients are less likely to receive chemotherapy, similar to randomized trials. Therefore, in a real-world population, these patients decrease the overall prognosis of individuals who do not receive chemotherapy, which may have inflated the difference in overall survival between the chemotherapy-treated and non-treated groups in this study. We consider our population as unselected while our patients were treated in a referral center. The mean age of patients treated the Institut Curie was 57.9 years (S.D. = 12.2), which is younger that mean age of breast cancer in USA or France (61 years) [17] [18]. However, the distribution of tumors’ characteristics in those patients treated in a referral center was representative of that observed in the breast cancer population as a whole [19] [20].

OS and DDFS improved over time (Table 3). It is difficult to determine the role of advances in therapeutics and screening (increasing the incidence of tumors discovered at an early stage) in a real-world population. Some studies attempted to estimate the impact of treatments and screening on breast cancer survival. Using 7 different modeling approaches, Berry *et al*. found that screening could explain 28% to 65% of the improvement in survival; 35% to 72% of the improvements were attributable to adjuvant treatments [21]. Another before/after study (1981–1984/1990–1994) estimated that one-third of the improvement in survival may be due to earlier tumors diagnosis, and the remaining two-thirds may be due to the adjuvant treatments [22]. However, the former study was based on models; the second was based on a small sample of patients. Additionally, the authors did not specify the role of chemotherapy alone. Our population-based study assessed the benefit of chemotherapy and confirmed that it provides a DDFS advantage primarily in the first years of follow-up (no additional benefit between 5 and 10 years, Fig 2) [23].

There is no consensus on the optimal method to assess the impact of treatment in a clinical setting. We chose propensity scores to minimize confounding and indication bias between the treated and non-treated groups. Other methods are employed to avoid bias (such as multivariable regression restricted to a high-risk population or the instrumental variable method [24]); however, none of these fully resolve the problem [25]. Our results obtained with the Cox multivariate model and with the propensity score matching method were similar, but the latter method could be used to assess whether the benefit occurs in the first years of treatment or thereafter. However, with the propensity score matching method, most of the patients who are excluded from the analysis exhibit low or high treatment probability. Our results reflect the average benefit of adjuvant chemotherapy in a population; however, this population likely excluded the patients who were unlikely to benefit or would certainly benefit from this treatment. We could not clarify the effect of chemotherapy in breast cancer subgroups because subgroups were not defined over the whole period at the time when patients were treated.

## Conclusions

In invasive breast cancer, adjuvant chemotherapy reduced the relative risk of death by 25% and the relative risk of distant metastasis by 18% in this study. After randomized trials, this study confirms the magnitude of the impact of chemotherapy in a real-world population and demonstrates the level of overtreatment. It advocates for better tumor evaluation and personalized treatment. The effect of chemotherapy in subgroups related to patient characteristics, tumor burden or tumor biological features remains to be clarified.
